# Every Day Practice

**Published:** 1886-07

**Authors:** J. Taft


### THE DENTAL REGISTER.

VOL. XL.] JULY, 1886. [No. 7.

### COMMUNICATIONS.

### Every Day Practice.

BY J. TAFT.

(Read before the Mad River Dental Society.)

What does it mean? What was in the mind of the committee presenting this subject?

Without knowing this definitely, I will venture a few thoughts in reference to "Every Day Practice;" and the first is in reference to the operator himself.

What is the responsibility of the dentist as he goes forth from day to day in the exercise of his vocation? Is it merely to derive some emolument and some advantage for himself? Doubtless this may enter into his daily work in a sense, but there is a higher and more responsible duty devolving upon him than this.

The welfare, the comfort, and the health of those who are in his charge, depend upon what he may do, and how his work is performed. The right performance of his duty to others constitutes the burden of his responsibility. No one ever experiences a great and deep sense of responsibility merely in making dollars or building up a reputation. These things will usually take care of themselves if our highest duty to others is fulfilled. If then, we day by day come upon important, responsible and difficult work, what preparation shall we make for its accomplishment? And here first, we may well consider the physical and mental condition of the operator. In this respect, almost everyone may be well or ill prepared, depending largely upon himself. It is the duty of the dentist as well as the physician—pre-eminently the duty of these, to maintain his health and strength in the most perfect state, in order that he may bring to his work his best skill and endeavor. This, his patient has a right to expect, and even to demand. It is, therefore, the duty of every dentist, to maintain his physical health and mental equilibrium in the best possible condition. All those practices and habits calculated to enfeeble or injure these should be avoided. It should be his daily study and effort to maintain his own health and general condition in the most perfect state, and give attention to those things that will best sustain him in his work.

Daily practice, especially the operative part, is laborious and exhausting, in many cases greatly so. The dentist should secure for himself an adequate amount of rest, diversion, exercise, fresh air, and sunlight; enough of these to restore his wasted energies. No specified amount of any or all of these can be prescribed for general adoption; some will require more than others. A cultivated intelligence must determine this point.

Change of thought and attention is a very important factor in maintaining the best possible state of health. The person who permits the mind and attention to settle down and follow one direction, fixed upon one object without change, ere long becomes enfeebled and exhausted and the mind reacts adversely upon the body. Those who can make a complete change of thought, turning quickly and wholly from a given object, and fix upon another, and so change from time to time, will be in the best condition.

It is one of the great embarrassments in the practice of our profession, that so many are out of proper condition for the performance of their work; and this ill condition is usually quite as well recognized by the victims and perhaps better than by any one else. Perhaps we have all had experience from which we ought to have learned far more than we have in this direction.

Coming to our work with a sense, perhaps, of lassitude, a weariness and indisposition to work, which may be physical only, or may be mental alone, or may be both, how often is it for hours, and it may be for a day, that we find things going wrong? Nothing seems to come out as we would have it; even our instruments and appliances seem pervaded by a kind of willful obstinacy, and refuse to perform their accustomed work rightly. It is a matter of serious consideration how far the flagging powers should be forced to work under such conditions. The best interest of the patient would suggest that work cease at once, and that only the best power and skill of the operator should be employed in the discharge of his duty.

Not only in these respects should the dentist have care, but his attention should ever be directed to having all the surroundings and every appliance of the most acceptable and efficient character. The work of the dental operator is always to say the least attended with discomfort, and in most cases with more or less of actual pain and suffering by the patient. It is the duty of the operator to render these as slight as possible. This relief cannot come, however, from feeble, faulty work; this should be sufficiently thorough to secure the best possible results.

The general bearing and sympathy of the operator will have much to do in modifying the condition of the patient for whom the work is done.

The instruments should be thoroughly adapted to the purpose for which they are used and employed. They should be in perfect condition, and used with dexterous skill. Every movement should produce its intended results; no false or faulty motions made.

Useless paraphernalia should be discarded, but every thing of real value for any and all cases should be at hand, and in condition for use.

Some are disposed to take up and immediately adopt every new thing that is presented, whether meritorious or not, and discard many good things simply because they are old. Upon the other hand some refuse the new, holding on to the old, and generally they are left far behind. A wise and discriminating judgment will direct aright in these matters.

One very common fault in the routine of daily practice is a neglect of details, a want of careful attention to little matters. This may have reference to the instruments and appliances that are in constant use, and it may go further and pertain to all the appointments of an office. Thorough system and arrangement should be observed in all things. The instruments and appliances for every case that may present should be at hand and in perfect condition.

A very common fault is found in a want of thorough care and attention to the ordinary every day operations. Some are accustomed to bestow great care and their best efforts upon the exceptionally difficult cases. This special attention to these cases is right, but the same care should be given to the common operations. How often is it that work is hurriedly and carelessly performed; manifest defects permitted to remain, which will ultimate in failure of the work. No one should permit himself to be hurried and pressed through his work. Due deliberation should be had in all cases. Only in this way can the best efforts be exercised and the best results secured.

In conference recently with a prominent member of our profession, one who as an operator has worked his way to the top, he remarked: "I never let an operation go from my hands in which, by close examination, I can find a fault or a flaw." And that man stands to-day where any member of our profession might be proud to stand.

The man who resolves to do the very best for every case will usually have to fight his way against many antagonisms.

Some of these are the undue desire to make money at once and rapidly. Another is an impaired physical condition. Another is a want of earnest disposition to do the best thing in all cases. Another will be found in the ignorance and want of appreciation of those whom he serves. This is exhibited in the want of a proper estimate of the value of the teeth as well as a want of faith in the ability of the operator. These things in some cases stand so prominently as to be very discouraging, and to turn many a one from the right way.

It is very probable that the committee who presented this subject desired that on the part of the members there should be presented an array of peculiar and instructive cases, but I have chosen to present these general thoughts on practice, leaving the presentation of cases and office instructions to the hands of others.

